# Malaria's association with climatic variables and an epidemic early warning system using historical data from Gezira State, Sudan

**DOI:** 10.1016/j.heliyon.2019.e01375

**Published:** 2019-03-21

**Authors:** Hamid H. Hussien

**Affiliations:** Department of Mathematics, College of Science and Arts, King Abdulaziz University, P.O. Box 344, Rabigh 21911, Saudi Arabia

**Keywords:** Infectious disease

## Abstract

Malaria is a major public health problem in Sudan. Climatic variability is the main risk factor for seasonal and secular patterns of *P. falciparum* malaria transmission in Gezira state. The purposes of this study is to (1) develop thresholds for action in a malaria epidemic early warning system using three traditional statistical methods including the mean number of malaria cases + 2 standard deviations (SD), percentiles over the median (medium + upper third quartile), and the cumulative sum over prior 10 years (C-SUM) and (2) explore to what extent the climate variability affects malaria transmission. Pearson's correlation coefficient for malaria incidence and rainfall, maximum temperature, relative humidity, and the Blue Nile River level was statistically significant (p < 0.05). However, there was an insignificant correlation between the number of malaria cases and the minimum temperature. Furthermore, the number of cases in 2015 was significantly higher than expected. An evaluation and comparison of the statistical methods for the early detection of malaria showed that there was a considerable variation in the number of cases exceeding an epidemic alert threshold.

## 1 Introduction

Malaria is one of the leading causes of illness and death and the world's most prevalent vector-borne disease [1, 2]. Overall, 90% of these deaths occur in sub-Saharan Africa, and the rest happen in Latin America and Asia. Although has been greatly reduced globally [3], malaria remains a main contributor to morbidity and mortality. In Sudan, malaria is a major health problem. The entire population is at risk of malaria epidemics with a very high burden on government and population [4]. According to the latest data, in 2016, about 37 million people were at risk of the disease and an estimated of 4000 deaths occurred [5]. *Plasmodium falciparum* is responsible for approximately more than 90% of cases [6, 7].

Climatic variability is thought to have a direct impact on the epidemiology of many vector-borne diseases [8]. It plays an important role in the transmission of malaria [9]. In particular; temperature, rainfall, and relative humidity are the most important variables in creating suitable conditions for malaria transmission. Temperature also plays a key role in malaria transmission as it regulates the development of mosquito larvae and affects the growth from egg to adult mosquito, even parasites [10, 11]. Rainfall also creates suitable conditions that allow sufficient surface water for mosquito breeding sites. Relative humidity also affects the survival rate of mosquitoes, as they cannot complete their transmission cycle under relative humidity less than 60% [11]. Floods may also lead to an increase in vector-borne diseases through the expansion in the number and range of vector habitats. Malaria epidemics in the wake of flooding are a well-known phenomenon in malaria-endemic areas worldwide [12]. This is especially true in Sudan where epidemic outbreaks have occurred along the Blue Nile River margins [13].

The implementation of malaria early warning systems is important to prevent and control malaria epidemics. The WHO has promoted it as an essential component of epidemic risk management [13]. The significance of the use of climatic factors in the development of malaria early warning systems in Africa has been demonstrated by several authors [13, 14, 15, 16]. Varying statistical methods used for malaria early warning systems may yield different results. In Ethiopia, Abeku et al. [17] and Teklehaimanot et al. [18] found that simple malaria epidemic detection methods worked best. There are presently no predictive models in the literature for the early detection of malaria epidemics or the monthly epidemic threshold for malaria incidence in Sudan.

## 2 Material and methods

In this study, we examined the association between climatic variability and the number of monthly malaria transmission in Gezira, Sudan and determined the threshold levels to trigger early warning for malaria outbreaks.

### 2.1 Study area

Gezira lies between the Blue Nile, the main river of the Nile, and the White Nile in the east-central region of Sudan. It has an area of 27,549 km^2^ and a population of 5,096,920. Gezira is subdivided into seven localities: El Gezira East, ElKamlin, El Hasaheisa, Um Algora, Wad Madni AlkObra, South Aljazeera, and El Managil. Gezira has a tropical climate characterized by a rainy season from June through December and a dry season from January through May. The mean annual maximum temperature ranges from 30°Cto 43 °C, and the minimum temperature ranges from 11 °C to 27 °C. The main activities in Gezira are agriculture; approximately 92% of the total area is agricultural land.

Malaria in Gezira is stratified as mesoendemic to hyperendemic, with markedly seasonal and intense transmission rates during the rainy season [19]. It occurs every year, with at least one peak, from August to December with *P. falciparum* as the predominant species [20], and *Anopheles arabiensis* are main mosquito [21]. The transmission of malaria is seasonal and depends on rainfall beginning in June. The recent Malaria Indicator Survey in Gezira showed that the malaria parasite prevalence among fever cases was in the category of 0–5 [22].

In 1974–1975, Gezira was affected by serious malaria epidemics. As a result, the endemicity and epidemicity of malaria in the state were of concern to health workers at the local, national, and international levels during the 1980s and 1990s. These epidemics led to a widespread control program, the Blue Nile Health Project, in 1975 with the assistance of the WHO, the World Bank, Kuwait, Japan, and the US government. Malaria control is the objective of this program, it has been successfully controlled for ten years, and the prevalence of the disease has decreased from 25% to less than 1%. The Blue Nile Health Project has been beset by many difficulties due to the problems associated with mosquito resistance to insecticides and parasite resistance to anti-malaria drugs [23] in addition to the discontinuation of external funding in 1989. Consequently, severe outbreaks occurred as dramatic epidemics in 1990, 1993–1994, 1995, and 2003–2004. The main factors known to influence these epidemics are climatic (heavy rain and floods), drought and famine, resistance of *P. falciparum* to antimalarial drugs, the movement of populations from low to high endemic areas, instability in bordering countries and the influx of refugees, and the establishment of large agricultural projects [24].

### 2.2 Data collection

In Sudan, malaria cases are reported to the Federal Ministry of Health from health centers/units and hospitals. In Gezira, cases are usually laboratory confirmed in urban centres and clinically diagnosed in rural areas [22]. In this study, monthly malaria case data from January 2007 to December 2016 were obtained from the Federal Ministry of Health. During the same period, monthly climate data, including the maximum temperature, minimum temperature, relative humidity, and rainfall were obtained from Sudan's metrological service, and Blue Nile water levels were obtained from the Ministry of Water Resources, Irrigation, and Electricity.

### 2.3 Statistical analysis

#### 2.3.1 Mean number of malaria cases + 2 standard deviations

Cullen et al. [25] He used a historical monthly data from the preceding years from which the epidemic years were excluded first proposed this method. Here, the epidemic threshold was defined as the mean number of malaria cases plus 1.96*SD. The WHO later modified this method in Africa by rounding up the standard deviation from 1.96 to 2 using at least time series of data for 5 years and excluding the epidemic years. This method was applied in Sudan [26] and Madagascar [27]. While it has the advantage of extreme simplicity, the SD is unacceptably high. In this paper, we were calculated the mean and standard deviation after deleting the extreme value of malaria cases for each month. The epidemic is tentatively flagged if the monthly incidence exceeds the mean number of malaria cases plus 1.96*SD.

#### 2.3.2 Percentiles over the median

This method has been implemented for the detection of highland malaria epidemics in Ethiopia [28] and Uganda [29]. The expression “normal epidemic channel” has been used by World Health Organization to describe the normal seasonal pattern of malaria in an area. In this method, the upper limit of the normal channel is the monthly third quartile from a consecutive 5–10 year series of monthly data. If the cases exceeded the third quartile in a certain month, then the epidemic is declared [23]. For this method, the 75^th^ percentile is a cut-off value beyond which an epidemic is flagged.

#### 2.3.3 The cumulative sum method

This method was developed by the Centers for Disease Control and Prevention [30]. The cumulative sums (C-SUM) determine the expected number of malaria cases using the average over three months (including the previous and following month) for five years. For calculating the expected number of cases for March 2012, we calculate the average of February, March, and April admissions from 2007 to 2011. An alert threshold is defined as the upper limit of a 95% confidence interval for the expected number of cases.

#### 2.3.4 Descriptive statistics

Descriptive statistics include the mean value, the 5% trimmed mean, the interquartile range (IQR) (which contains the middle 50% of the data and is unaffected by extreme values), and the median (which has 50% of the data below), were calculated for all of the variables during the low and high transmission seasons. In addition, 95% confidence interval (CI) around the mean number of malaria cases each month was constructed using the following formula

(1) $$95\%CI=\bar{X}\pm Z_{\alpha /2}\left(\frac{s}{\sqrt{n}}\right)\text{,}$$

Where $\bar{X}$ is the mean number of malaria cases in the month, *s* is the standard deviation, *n* is the sample size, and $Z_{\alpha /2}$ is the value of normal distribution with the tow tail level of significance α = 0.05. A one-way analysis of variance was used to test if there was a statistically significant difference in the number of malaria cases with respect to the months. In addition, a multiple comparison (known as post hoc) test was conducted to determine where the monthly number of malaria cases differed.

## 3 Results

### 3.1 Association between climate variability and malaria cases

A statistical data analysis was performed using Excel. We used the monthly incidence of malaria in Gezira as an outcome variable, and climatic variables such as the monthly mean maximum and minimum temperatures, the monthly mean relative humidity, the total monthly amount of rainfall, and the Blue Nile's monthly mean water level were independent variables. Spearman's correlation analysis was conducted to examine the relationship between the monthly climatic variables and the incidence of malaria.

Table 1 presents the correlations between all of the variables considered in this study. The results suggest that the number of malaria cases has a significantly negative linear correlation with the maximum temperature and a significantly positive linear correlation with the relative humidity, the amount of rainfall, and the Blue Nile's level (p < 0.05). However, there was a non-linear relationship between the number of malaria cases and the minimum temperature.

**Table 1:** Correlation coefficients between climatic factors and malaria cases.

	Max. temperature	Min. temperature	Relative humidity	Rainfall	Blue Nile level
No. of malaria cases	−0.258^(∗∗)^	0.003	0.435^(∗∗)^	0.292^(∗∗)^	0.411^(∗∗)^
Max. temperature		0.748^(∗∗)^	−0.294^(∗∗)^	−0.213^(∗)^	−0.170
Min. temperature			0.334^(∗∗)^	0.327^(∗∗)^	0.398^(∗∗)^
Relative humidity				0.717^(∗∗)^	0.866^(∗∗)^
Rainfall					0.711^(∗∗)^

**Correlation is significant at the 0.01 level.

*Correlation is significant at the 0.05 level.

**Fig. 1:**
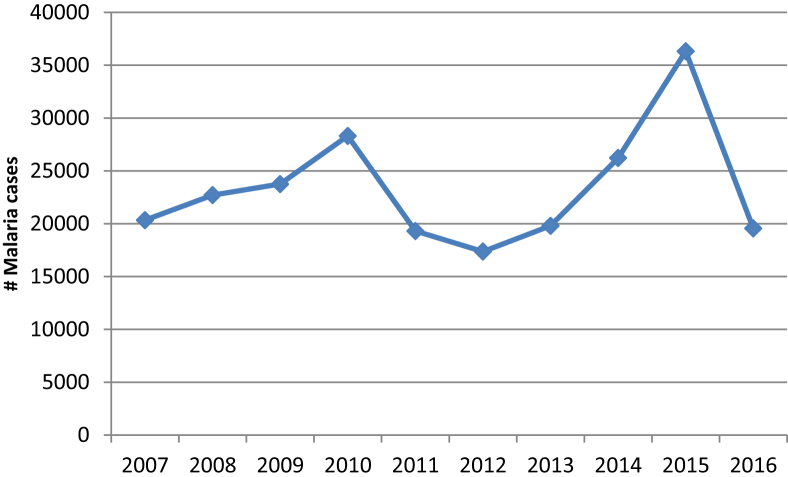
Annual incidence of malaria in Gezira state, Sudan, 2007–2016.

Fig. 1 represents the annual incidence of malaria in Gezira during the study period from 2007 to 2016. The total number of cases was 233,960, ranging from 17,363 in 2012 to 36,322 in 2015, with a monthly mean of 1,947 and a standard deviation (SD) of 778. The annual mean number was 23,369. The highest number of malaria cases was 36,322 reported in 2015.

**Fig. 2:**
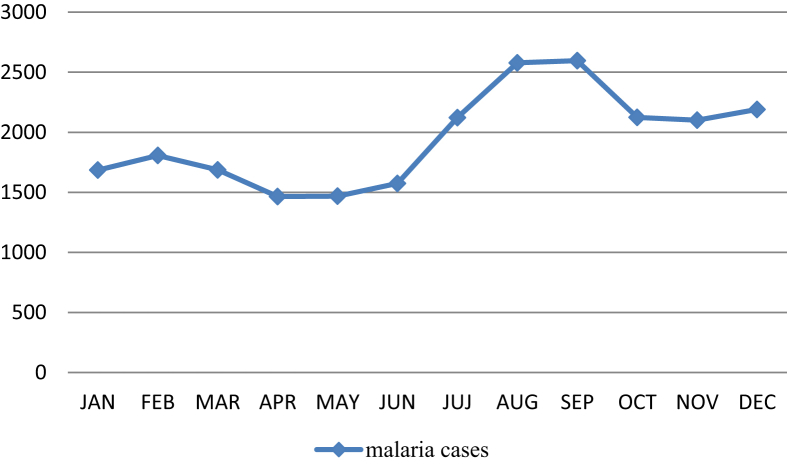
Monthly mean numbers of malaria cases in Gezira state (2007–2016).

Fig. 2 plots the monthly mean numbers of malaria cases in Gezira State during the study period. The variation in malaria cases during the year was confirmed and the graph indicates that there was a monthly variation in malaria cases in the state with a peak time from July to December, although malaria occurred in approximately every month of the year. The analysis of variance test showed that the mean number of malaria cases was significantly different for at least one of the months in the year (F_11_, 108 = 3.004, p < 0.01). Following up with multiple comparisons (or post hoc) tests (Table 2), the result shows that the data provide statistical evidence that there was a difference in the mean numbers across the months of the year. Accordingly, the tests divide data into two groups that there is no significant difference between them. Group 1 includes the months January to June, it is noted here as low transmission season, Group 2 includes the months July to December, and it is note here as high transmission season.

**Table 2:** Result of Multiple comparisons tests for the monthly mean number of malaria cases.

Month	Group 1 (P-value = 0.06)						Group 2 (P-value = 0.19)					
	APR	MAY	JUN	MAR	JAN	FEB	NOV	JUJ	OCT	DEC	SEP	AUG
# Incidences	1465	1468	1574	1687	1685	1807	2101	2122	2124	2190	2596	2578
Subset for alpha = .05												

Table 3 presents the summary statistics for the two transmission seasons (high transmission and low transmission) of malaria cases, the levels of rainfall, the relative humidity, the maximum and minimum temperatures, and the Blue Nile's level. During the high transmission season, the average monthly number of malaria cases was 2010 (SD = 568) with a median of 1,991 (IQR: 678) compared to an average monthly number of malaria cases 1,614 (SD = 450) with a median of 638 (IQR: 638) during the low transmission season. This confirms the hypotheses of a strong difference in malaria infection and disease between the dry and rainy seasons. The monthly mean values of the maximum temperature, minimum temperature, relative humidity, the amount of rainfall, and the Blue Nile's level were 36.16 °C (SD = 2.07), 20.79 °C (SD = 3.15), 54.03 (%) (SD = 15.05), 42.05 mm (SD = 55.29), and 13.79 mm (SD = 2.57), respectively, between 2006 and 2015, during the high transmission season compared to 38.18 °C (SD = 3.78), 19.69 °C (SD = 4.67), 29.67 (%) (SD = 8.28), 5.65 mm (SD = 13.94), and 10.62 mm (SD = 0.73) during the low transmission season in the same period.

**Table 3:** Descriptive statistics for monthly malaria incidence and climatic factors.

Variable	High transmission season^a^				Low transmission season^a^			
	Monthly mean (SD)	5% Trimmed mean	95% CI	Median (IQR)^b^	Monthly mean (SD)	5% Trimmed mean	95% CI	Median (IQR)^b^
Malaria cases	2010 (568)	2015	1852–2168	1991 (678)	1614 (450)	1585	1498–1731	638 (638)
Minimum temperature (°C)	20.39 (3.15)	21.22	20.25–21.86	21.95 (4.98)	19.69 (4.67)	18.60	17.38–19.86	18.50 (3.38)
Maximum temperature (°C)	36.16 (2.07)	36.82	36.22–37.44	36.80 (3.38)	38.18 (3.78)	37.72	36.52–38.74	38.2 (7.67)
Relative humidity (%)	54.03 (15.05)	51.44	48.41–55.53	26.00 (49.50)	29.67 (8.28)	27.36	25.59–19.77	27 (13)
Rainfall (mm)	42.05 (55.29)	32.28	25.71–50.67	10.66 (56.80)	5.65 (13.94)	1.26	0.13–7.4	0.00 (0.01)
Blue Nile level (mm)	13.79 (2.57)	13.39	12.81–14.05	13.29 (4.21)	10.62 (0.73)	10.44	10.29–10.63	10.44 (0.71)

a For malaria incidences, high transmission season was defined from 1July to 31 December, opposite to low transmission season defined from 1 January to 30 June of each year.

b IQR: Interquartile range.

### 3.2 Determining thresholds for early warning

To detect an epidemic, we estimated the expected number of cases for the state and time period. The three methods were adopted to identify the month with more malaria cases than expected. According to the data analysis, the thresholds of the three methods are shown in Tables 4, 5, and 6.

**Table 4:** Mean number of malaria cases ± 2 (SD).

Threshold	Jan.	Feb.	Mar.	Apr.	May	Jun.	Jul.	Aug.	Sep.	Oct.	Nov.	Dec
Upper limit	2219	2867	2761	2347	1946	2212	2637	3597	3435	3411	3264	3180
Lower limit	867	747	613	583	990	936	1269	1117	1239	427	436	728

**Table 5:** First quartile, median and third quartile alert thresholds.

Threshold	Jan.	Feb.	Mar.	Apr.	May	Jun.	Jul.	Aug.	Sep.	Oct.	Nov.	Dec
1^st^ quartile	1215	1219	1170	1171	1276	1252	1653	1948	1940	1450	1415	1700
Median	1595	1862	1662	1408	1512	1601	1953	2316	2334	1966	1999	2130
3^rd^ quartile	1936	2104	2019	1591	1589	1856	2389	3219	3191	2686	2542	2618

**Table 6:** The cumulative sum (C-SUM) method and C-SUM + 1.96 SD for each month.

Threshold	Jan.	Feb.	Mar.	Apr.	May	Jun.	Jul.	Aug.	Sep.	Oct.	Nov.	Dec
C-SUM	1500	1439	1378	1283	1252	1371	1549	1762	1701	1612	1505	1503
C-SUM + 1.96 SD	2581	2477	2430	2148	1719	1997	2914	3798	3469	3332	3243	3161

Table 4 illustrates the most epidemic surveillance techniques, the mean number of malaria cases ± 2 (SD), which identify those points in a malaria time series that occur outside the 95% confidence intervals of a normal distribution determined from the history of malaria cases.

Table 5 illustrates the first quartile, the median, and the third quartile. The third quartile value pinpoints the level above which the possibilities of an epidemic occur.

**Fig. 3:**
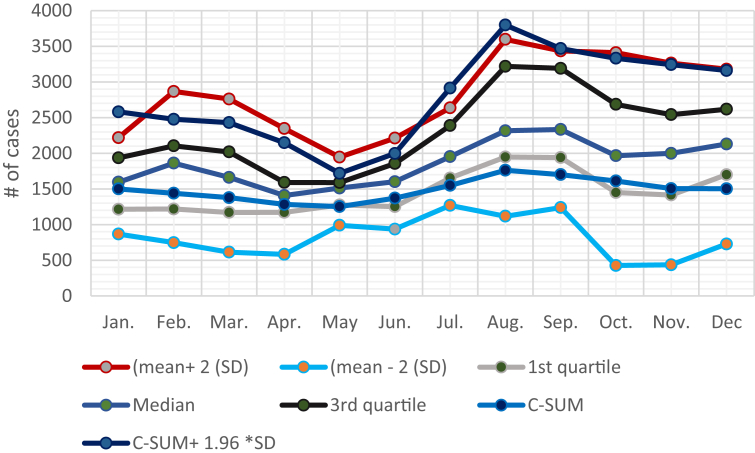
Threshold levels trigger early warning for malaria outbreaks.

Table 6 demonstrates the C-SUM method and C-SUM + 1.96 SD for each month. The C-SUM method is refined by adding the 95% confidence interval (1.96 times the standard deviation) for each month of the threshold. These values can be used as the threshold above which an epidemic alert is triggered. In general, the results suggest that the number of malaria cases in January and July (2,581 and 2,914, respectively) were the most significant and higher than expected. The alert threshold is now determined and presented in Fig. 3. An epidemic occurs if the number of malaria cases rises above the upper limit of normal; so that an epidemic should be declared and clear processes for action should be taken.

**Fig. 4:**
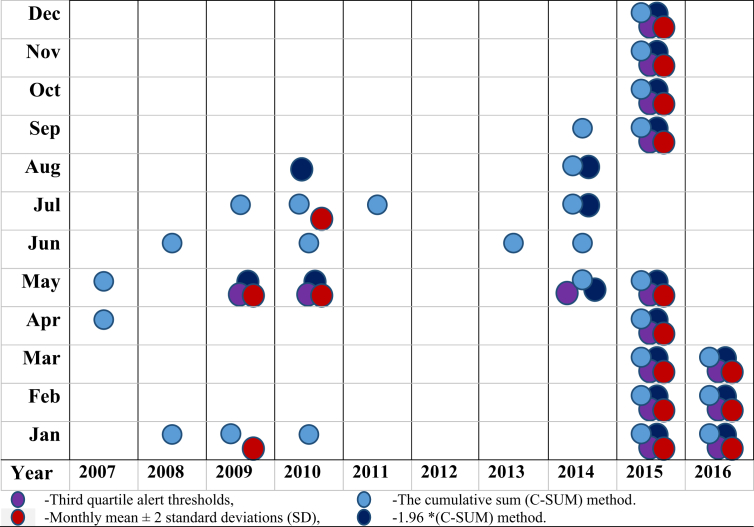
Flowchart of Method of analysis evaluated for the use of early warning for malaria outbreaks.

The results of the malaria early warning system described in this paper using three simple statistical methods are summarized and presented in Fig. 4. It is easily seen that the numbers of malaria cases in January were far above the threshold signaling an epidemic in 2008, 2009, 2010, 2015, and 2016. Epidemics occurred in February and March of 2015 and 2016. The numbers of malaria cases in October were far above the threshold only in 2015. In general, there were considerable variations in the number of cases exceeding an epidemic threshold during the study period. The study found 51 outbreaks occurred for which the system would have provided early warnings. In 2015, there were 9 alerts, ample evidence that an outbreak was occurring, even considering the drop in May, June, and July. The results also showed that the most number of alerts was set using the C-SUM method, 28 alerts, followed by the 1.96 *(C-SUM) method, 18 alerts, followed by the monthly mean ± 2 (SD) method, 11 alerts. The third quartile methods showed only 4 alerts. The mode of the three methods is the C-SUM method; therefore, we suggest it as the most responsive technique for a malaria early warning system in Gezira.

## 4 Discussion

There is presently strong interest in assessing the relationship between climate variability and malaria incidence. This study investigated the association between climatic factors and the number of malaria cases in Gezira, Sudan, using Pearson's correlation coefficient. We found that the number of malaria cases has a significantly negative linear correlation with the maximum temperature. This finding could be justified by increasing temperature that which could extinct the parasite in the vector [31]. This is in agreement with other results in the literature showing that increasing maximum temperatures limited malaria incidence. For example, Nkurunziza et al. found that the maximum temperature has been shown to have a negative effect on malaria incidence. This relationship could be explained by the interruption of mosquito development caused by high temperatures [32] and their inhibitory and lethal effects [14]. Specifically, it has been demonstrated that the proportion of parasites surviving decreases rapidly at temperatures over 32–34 °C [33]. The result also shows a significantly positive linear correlation with the relative humidity, the amount of rainfall, and the Blue Nile's level. However, there was a non-linear relationship between malaria incidence and the minimum temperature. We found a statistically significant increase in the rainfall, minimum temperature, relative humidity, and the Blue Nile's level during the high transmission season compared to the low transmission season. However, the maximum temperature was lower in the low transmission season than the high transmission season. The larger interquartile ranges of malaria cases, the monthly mean minimum temperature, relative humidity, and rainfall was detected in the high transmission season, showing larger differences in these variables during this season. Some studies of the effect of climate factors on malaria epidemics in Sudan suggests that higher temperatures and heavier rainfall encourage anopheles mosquitos, malaria carriers, to multiply and move further. Therefore, malaria cases are associated positively with temperatures as well as rainfall and flood events [23, 24]. In contrast, some studies in India failed to find a significant correlation between annual rainfall and malaria incidence [34, 35]. Likewise, a study in Kwa Zulu-Natal province in South Africa also failed to find such a correlation between the annual malaria incidence and rainfall. While, a significant positive correlations were found between the difference in successive 12 monthly (July to June, corresponding to the local malaria season) logarithmically transformed malaria case totals and summer (November to March) rainfall [36]. In Botswana, it is found that there is a positive correlation between annual malaria anomalies and December to February rainfall [29]. Our results are somewhat in line with those found in many African countries [32, 37].

In this study, we used four different statistical methods to calculate the alert thresholds for malaria epidemics in Gezira. This system is sufficiently simple to be applied at the lowest level of malaria control, reliable enough to indicate abnormal numbers of malaria cases in Gezira, and sensitive enough to provide timely warnings of impeding outbreaks. The prepared graph for the threshold levels of malaria outbreaks (Fig. 3), should be sent to the health unit director, who should thereafter be responsible for continuing to plot the actual monthly number of malaria cases, as soon as they become available, and immediately inform the regional heath director if the number of cases exceeds the normal threshold for a given month.

In July 2001, a weekly notification system was established in Sudan and an epidemic threshold was established for each state. The project considered various aspects of malaria including metrological data. Unfortunately, this system failed to properly detect small outbreaks during 2003. The problem was that the malaria managers at the state level did not carefully assess the data [38].

The early warning system described in this paper is only a first step in preventing malaria epidemics. Nevertheless, it provides an opportunity to change the outlook on malaria because early action might dramatically alter the course of events. However, warnings alone are insufficient. A full epidemiological investigation is required to assess the locality and extent of outbreaks, prioritize prevention, and control measures. Furthermore, developing the will and capacity to respond in a timely fashion to warnings of increases in transmission risk is the real test for malaria control organizations. A contingency plan for epidemic preparedness, forecasting, and rapid response is needed.

## 5 Conclusion

Over the past few decades, morbidity and mortality from malaria have increased public health problems in Gezira. Despite the substantial potential for malaria risk monitoring to help initiate malaria control efforts prior to epidemics, the control of the disease faces major difficulties due to the growing problems associated with mosquito resistance to insecticides and parasite resistance to anti-malaria drugs. Developing accurate predictions/alert thresholds of malaria outbreaks using climate/malaria data remains a challenge. However, these simple statistical methods for determining monthly epidemic threshold levels could provide tools for forecasting when there will be increased risks of malaria epidemics in Gezira. Comparing the methods we used in this study, the result (Fig. 4), suggests that the C-SUM method is the best technique to trigger early warning for malaria outbreaks in Gezira. The three methods can provide excellent indications of the beginning of outbreaks depending on the level and quality of surveillance, the degree of transmission, and the number of years of retrospective data available for analysis. Furthermore, this study established a significant association between malaria cases and the maximum temperature, relative humidity, amount of rainfall, and the Blue Nile's level. However, no significant association between malaria cases and the minimum temperature was found.

## Declarations

### Author contribution statement

Hamid H. Hussien: Conceived and designed the analysis; Analyzed and interpreted the data; Contributed analysis tools or data; Wrote the paper.

### Funding statement

This research did not receive any specific grant from funding agencies in the public, commercial, or not-for-profit sectors.

### Competing interest statement

The authors declare no conflict of interest.

### Additional information

No additional information is available for this paper.
